# Diagnostic performance of microRNA-29a in active pulmonary tuberculosis: a systematic review and meta-analysis

**DOI:** 10.1016/j.clinsp.2023.100290

**Published:** 2023-10-12

**Authors:** Xiaoying Li, Yuehong Xu, Pu Liao

**Affiliations:** a Department of Clinical Laboratory, The Affiliated Hospital of Southwest Medical University, Luzhou, China; b Department of Clinical Laboratory, Chongqing General Hospital, Chongqing, China

**Keywords:** Tuberculosis, microRNA-29a, Diagnosis, Meta-analysis

## Abstract

**Background:**

In recent years, more and more studies have shown that microRNA-29a (miRNA-29a) can be used as a potential biomarker for active tuberculosis, but the results of these studies are not consistent.

**Objective:**

To comprehensively evaluate the value of miRNA-29a in the diagnosis of active tuberculosis by meta-analysis.

**Methods:**

The databases of CNKI, WanFang, PubMed, The Cochrane Library, Web of Science and EMBASE were searched for relevant studies. Studies were screened strictly according to inclusion and exclusion criteria. QUADAS-2 scale was used to evaluate the quality of the included studies. Data were extracted and analyzed by Meta-DiSc 1.4 and Stata 16.0 software.

**Results:**

13 articles were included, including a total of 1598 subjects, including 872 active tuberculosis patients and 726 controls. The combined sensitivity and specificity of miRNA-29a in the diagnosis of active tuberculosis were 78 % and 76 %, respectively, and the area under the overall summary receiver operating characteristic curve was 0.8564.

**Conclusion:**

miRNA-29a can be used as a biomarker for the diagnosis of active tuberculosis.

## Highlights

• A meta-analysis was applied to analyze the performance of miRNA-29a in diagnosing active pulmonary tuberculosis.
• miRNA-29a can be used as a biomarker for the diagnosis of active tuberculosis.
• Tuberculosis burden and sample type of miRNA-29a may influence its diagnostic performance.

## Introduction

Tuberculosis (TB) is an infectious disease caused by *Mycobacterium Tuberculosis* (MTB), which is spread by droplets with pathogenic bacteria. The disease usually occurs in the lungs, but any extra-pulmonary site may be involved.^1^ Currently, nearly 25 % of patients with latent TB infection worldwide.^2^ Although patients with latent infection have no clinical symptoms, 5 %‒10 % of them may develop active TB and become a new source of infection.^3^ According to the World Health Organization, the global incidence of TB has seen a partial recovery after a sharp decline due to the impact of the novel coronavirus pneumonia pandemic. With some 6.4 million new TB cases reported globally in 2021 and over 1.6 million deaths, the global TB burden is still heavy.^4^ Diagnosing TB can be challenging because of its different clinical forms and disease severity.^5^ In order to realize the strategy of eliminating TB, it is the key to develop simple, reliable, and economical biomarkers to diagnose the disease.

MicroRNAs (miRNAs) are small regulatory non-coding RNAs with a length of about 22 nucleotides, which mainly play roles in the post-transcriptional regulation of gene expression,^6^ and are crucial for biological processes such as cell proliferation, differentiation, and apoptosis.^7^ Altered expression patterns of miRNAs have been demonstrated in multiple human diseases, and their dysregulation is an important factor in disease progression.^8^ The lungs have a very specific miRNA expression profile, and these miRNAs play undeniable roles in lung development and maintaining lung homeostasis. The changes in miRNA expression profile may be related to pathological processes in the lungs and lead to the development of various lung diseases, including mild inflammatory diseases to severe lung cancer.^9^^,^^10^ Garg et al.^11^ found that the serum miRNA-29a level in TB patients was significantly higher than that in healthy controls, indicating a good ability to distinguish TB patients from healthy people. The results of another study also showed that miRNA-29a could be used as a biomarker of active TB, and the expression level was related to clinical characteristics.^12^

In recent years, miRNA-29a has been widely studied in the diagnosis of active TB, but the results are inconsistent. This study aims to comprehensively evaluate the value of miRNA-29a in the diagnosis of active TB through meta-analysis, providing an evidence-based basis for further research and clinical application of miRNA-29a.

## Methods

### Search strategy

This meta-analysis was conducted according to the Preferred Reporting Items for Systematic Reviews and Meta-Analysis (PRISMA) guidelines 2020.^13^ The authors searched the literature on miRNA-29a diagnosis of active TB in CNKI, Wanfang, PubMed, The Cochrane Library, Web of Science, and EMBASE databases. The search terms were as follows: (“tuberculosis” or “active tuberculosis” or “mycobacterium” or “TB” or “ATB”) and (“microRNA-29a” or “miRNA-29a” or “miR-29a”). The retrieval time was from the establishment of each database to December 2022.

### Inclusion and exclusion criteria

The inclusion criteria were as follows: (1) Original studies assessed the accuracy of miRNA-29a in diagnosing active TB, (2) There were clear reference standards for the diagnosis of each individual, (3) Studies reported True Positive (TP), False Positive (FP), True Negative (TN), and False Negative (FN), or provided sufficient data to calculate them.

The exclusion criteria were as follows: (1) Reviews and conference abstracts, (2) The research was not aimed at active TB, (3) Not a diagnostic study, (4) The study was not focused on miRNA-29a.

### Data extraction

The authors read the entire content of each eligible article, and a researcher extracted the following data: the first author, country of publication, year of publication, sample type, TP, TN, FP, FN, and participant characteristics. All data were aggregated and processed in the form of characteristic tables. Another researcher checked the data for accuracy and completeness. Disagreements were resolved through discussion.

### Quality assessment

The authors assessed the quality and risk of bias of each study using the Quality Assessment of Diagnostic Accuracy Studies (QUADAS-2), which included four fields: patient selection, index test, reference standard, flow, and timing. The evaluation result was defined as “yes”, “no”, or “unclear”, with corresponding risk biases of “low”, “high”, and “unclear”.

### Statistical analysis

Meta-DiSc 1.4 and Stata 14.0 software were used for statistical analysis. The Spearman correlation coefficient between the logarithm of sensitivity and the logarithm of (1-specificity) was calculated to test the threshold effect. If *p <* 0.05, it indicates the existence of a threshold effect. Calculate the I^2^ index of sensitivity and specificity to evaluate heterogeneity caused by non-threshold effects between studies. *I*^2^ values above 50 % were considered significant heterogeneity, then a random effects model should be used to combine sensitivity, specificity, Positive Likelihood Ratio (PLR), Negative Likelihood Ratio (NLR), and diagnostic Odds Ratio (DOR). And further explored the sources of heterogeneity. Evaluated the overall diagnostic value of miRNA-29a using the overall Summary Receiver Operating Characteristic (SROC) curve. Finally, the Deeks' funnel plot asymmetry test was used to evaluate publication bias.

## Results

### Literature search

**Fig. 1:**
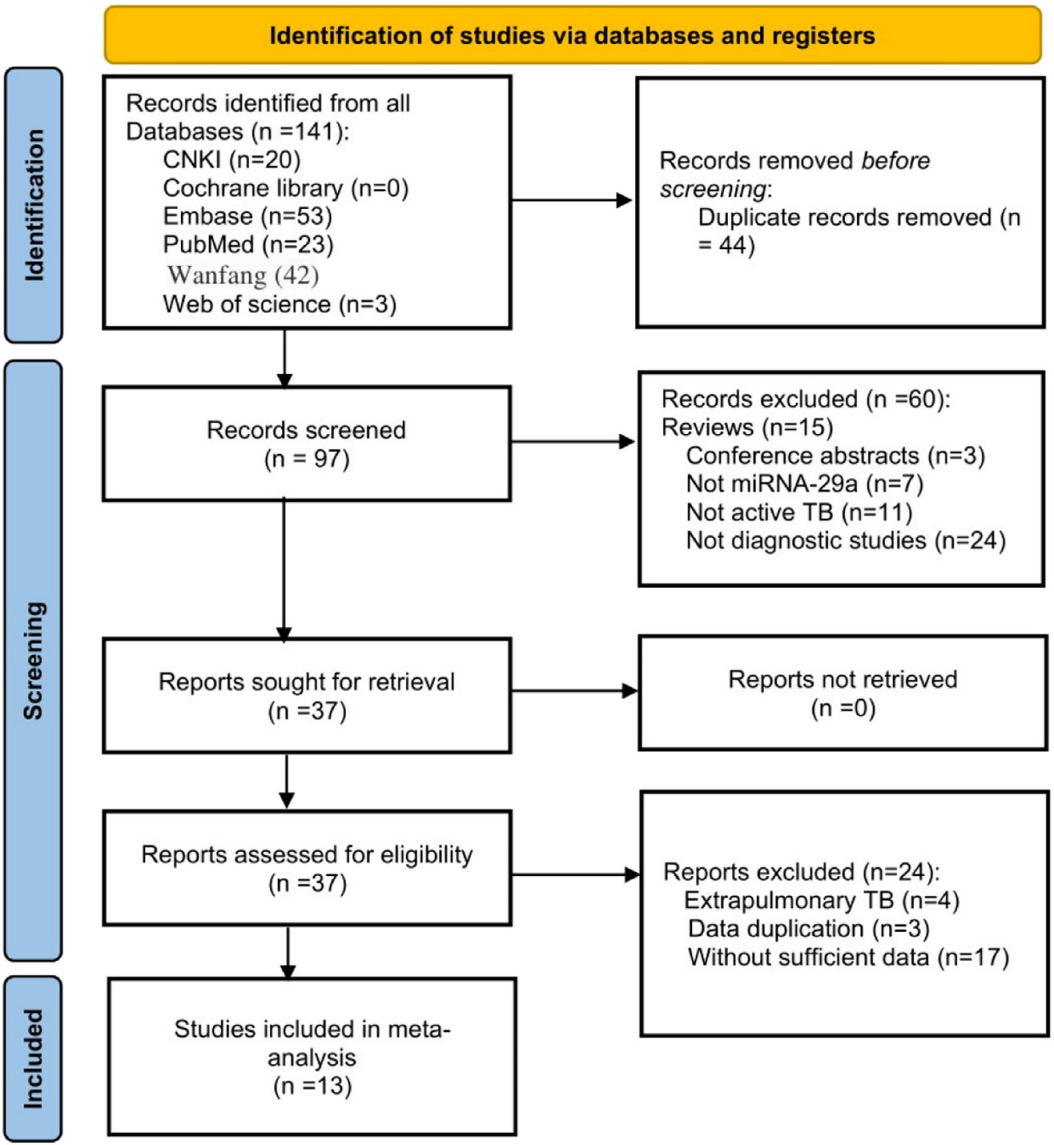
Flow chart of the study selection process.

After a preliminary search, a total of 141 records were found. The authors removed 44 duplicate articles and excluded 60 articles by reading titles and abstracts. Then carefully read the full text of the remaining 37 articles. Since extrapulmonary TB is generally not contagious, the authors excluded those articles, as well as articles with duplicate data and articles without sufficient data. Finally, 13 articles were included for analysis.^11^^,^^12^^,^^14^, ^15^, ^16^, ^17^, ^18^, ^19^, ^20^, ^21^, ^22^, ^23^, ^24^ The flow chart of the research search and selection is shown in Fig. 1.

### Study characteristics and quality assessment

**Fig. 2:**
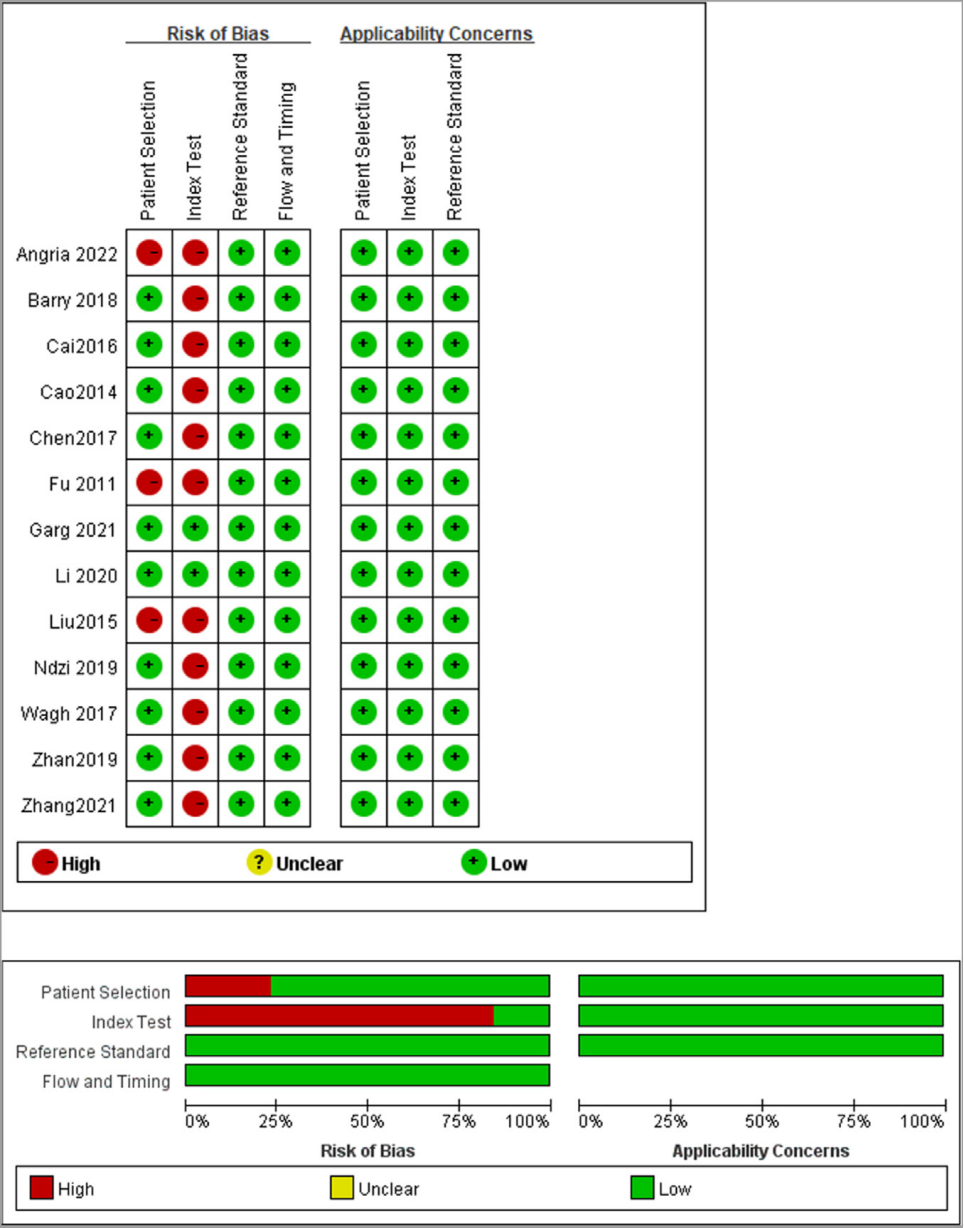
Quality assessments of the included studies.

The included 13 studies were published from 2014 to 2022 and were from China (8), India (2), Australia (1), Indonesia (1), and Cameroon (1), covering a total of 1598 subjects, of which 872 active TB patients and 726 controls. In all included studies, the diagnosis of TB was based on bacteriological, clinical, and radiological evidence, and miRNA-29a was determined by real-time quantitative Polymerase Chain Reaction. The characteristics of the included studies are listed in Table 1. The authors used the QUADAS-2 tool to assess the basis of patient selection, index test, reference standard, flow and timing, and the risk of bias of applicability concerns. The result was visualized, as shown in Fig. 2.

**Table 1:** Characteristics of the included studies.

Author	Year	Country	Age	TB burden	HIV status (n)	Sample	Dysregulated status	Case/Control (n)	TP	FP	FN	TN
Angria^20^	2022	Indonesia	Adult	High	NA	Blood	Up	50/30	43	8	7	22
Barry^14^	2018	Australia	Adult	Low	NA	Plasma	Up	100/100	37	4	63	96
Cai^21^	2016	China	Adult	High	Negative	Plasma	Up	30/30	24	9	6	21
Cao^18^	2014	China	Adult	High	Negative	Plasma	Up	40/60	27	15	13	45
Chen^22^	2017	China	Adult	High	NA	Plasma	Up	45/45	30	3	15	42
Fu^15^	2011	China	Adult + child	High	Negative	Serum	Up	30/30	25	6	5	24
Garg^11^	2021	India	Adult	High	NA	Blood	Up	30/30	24	5	6	25
Li^12^	2020	China	Adult	High	NA	Serum	Up	192/186	172	54	20	132
Liu^19^	2015	China	Adult	High	Negative	Blood	Up	60/20	42	6	18	14
Ndzi^16^	2019	Cameroon	Adult + child	Low	Positive (17)	Plasma	Up	62/42	50	12	12	30
Wagh^17^	2017	India	Adult	High	Negative	Serum	Down	30/30	29	16	1	14
Zhan^23^	2019	China	Adult	High	Negative	Plasma	Up	151/78	142	28	9	50
Zhang^24^	2021	China	Adult	High	Negative	Plasma	Up	52/45	35	9	17	36

Dysregulated status: miRNA-29a was up-regulated or down-regulated in each study.

### Diagnostic accuracy of miRNA-29a in active tuberculosis

**Fig. 3:**
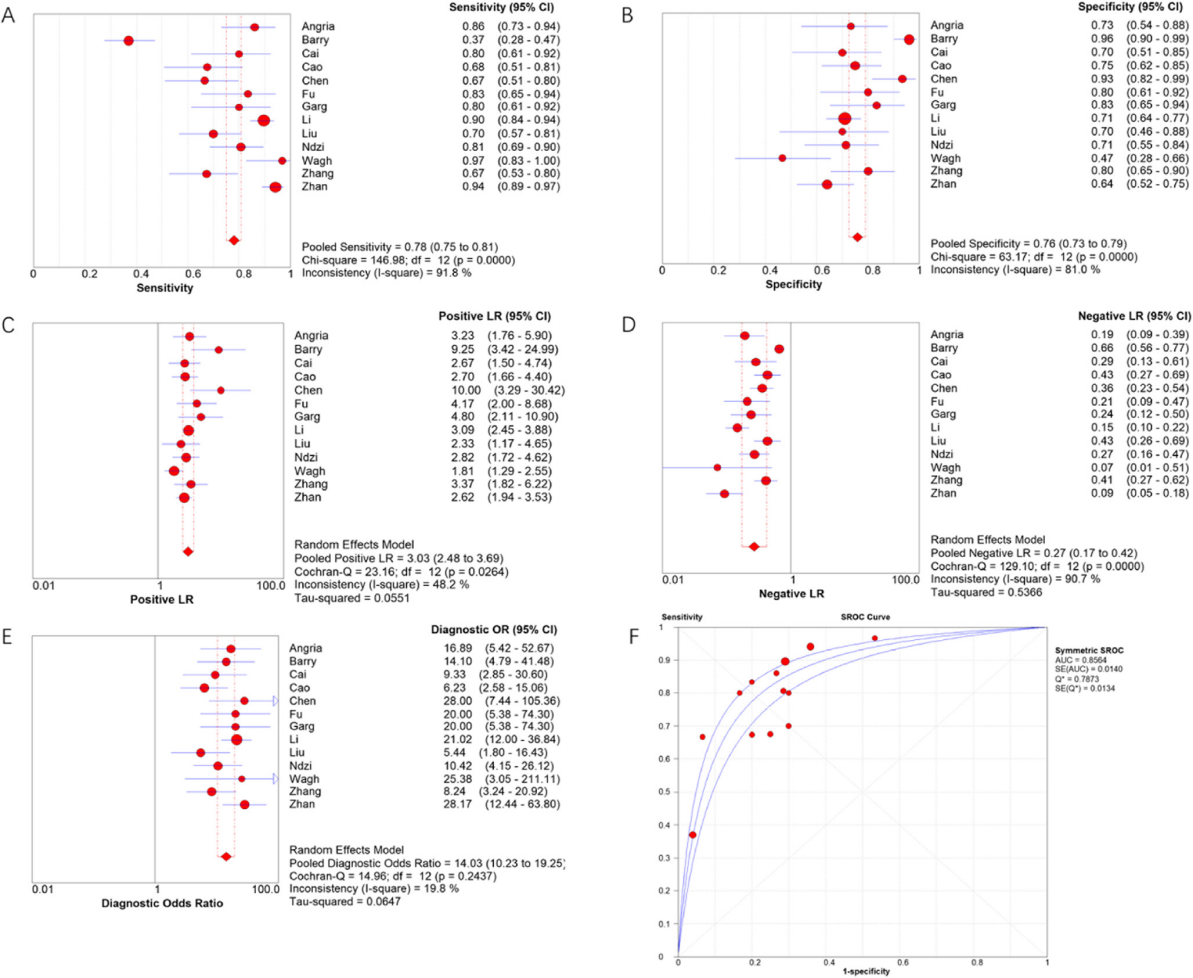
The forest plots of combined sensitivity (A), specificity (B), PLR (C), NLR (D), DOR (E) and SROC curve (F) for miRNA-29a to diagnose active tuberculosis.

Meta-DiSc software was used to analyze extracted data. The *I*^2^ of sensitivity and specificity were 91.8 % and 81.0 %, respectively, indicating significant heterogeneity. Therefore, the authors used the random effects model to combine diagnostic indicators. The combined sensitivity of miRNA-29a for diagnosing active TB was 0.78 (95 % CI 0.75‒0.81), the combined specificity was 0.76 (95 % CI 0.73‒0.79), the combined PLR was 3.03 (95 % CI 2.48‒3.69), the combined NLR was 0.27 (95 % CI 0.17‒0.42), and the combined DOR was 14.03 (95 % CI 10.23‒19.25). The area under the SROC curve was 0.8564. Forest plots of indicators and the SROC curve are shown in Fig. 3.

### Threshold effect and sensitive analysis

**Fig. 4:**
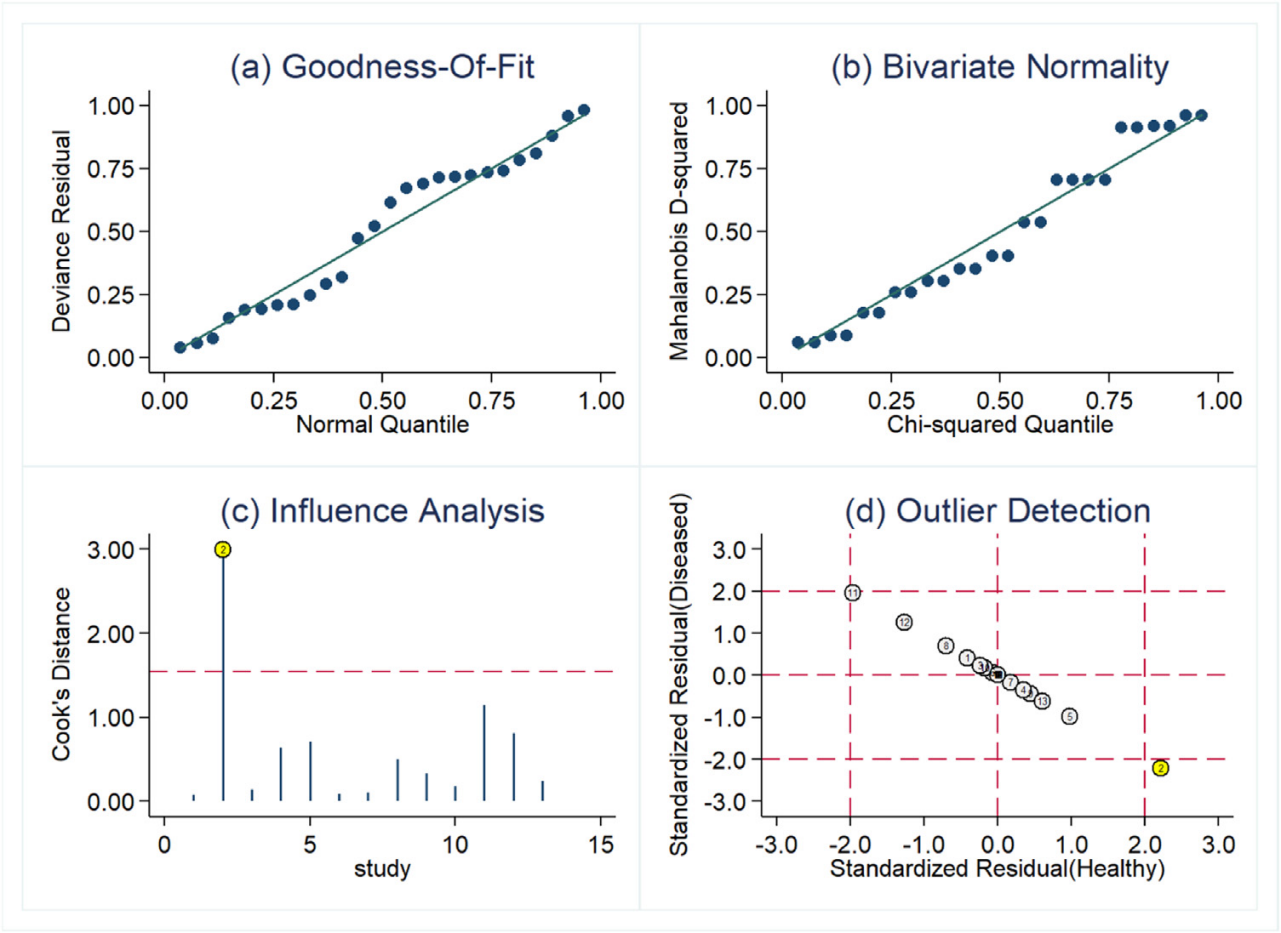
Sensitive analysis result.

Due to the heterogeneity that may be caused by the threshold effect and non-threshold effect, the authors used the Spearman correlation coefficient (0.730, *p* = 0.005, *p <* 0.05) to evaluate the threshold effect, and the result showed that there was a threshold effect in this study. Sensitive analysis was performed by Stata software. As shown in Fig. 4, one original study showed strong sensitivity. After excluding the study by Barry et al.,^14^ the combined sensitivity was 0.83 (95 % CI 0.80‒0.86), the combined specificity was 0.73 (95 % CI 0.69‒0.76), the combined PLR was 2.87 (95 % CI 2.41‒3.42), the combined NLR was 0.25 (95 % CI 0.19‒0.35), and the combined DOR was 13.96 (95 % CI 9.89‒19.70). Except for combined sensitivity, all other diagnostic indicators had decreased to varying degrees, indicating that there may be significant heterogeneity between this study and the other 12 studies.

### Subgroup analysis and meta-regression

**Fig. 5:**
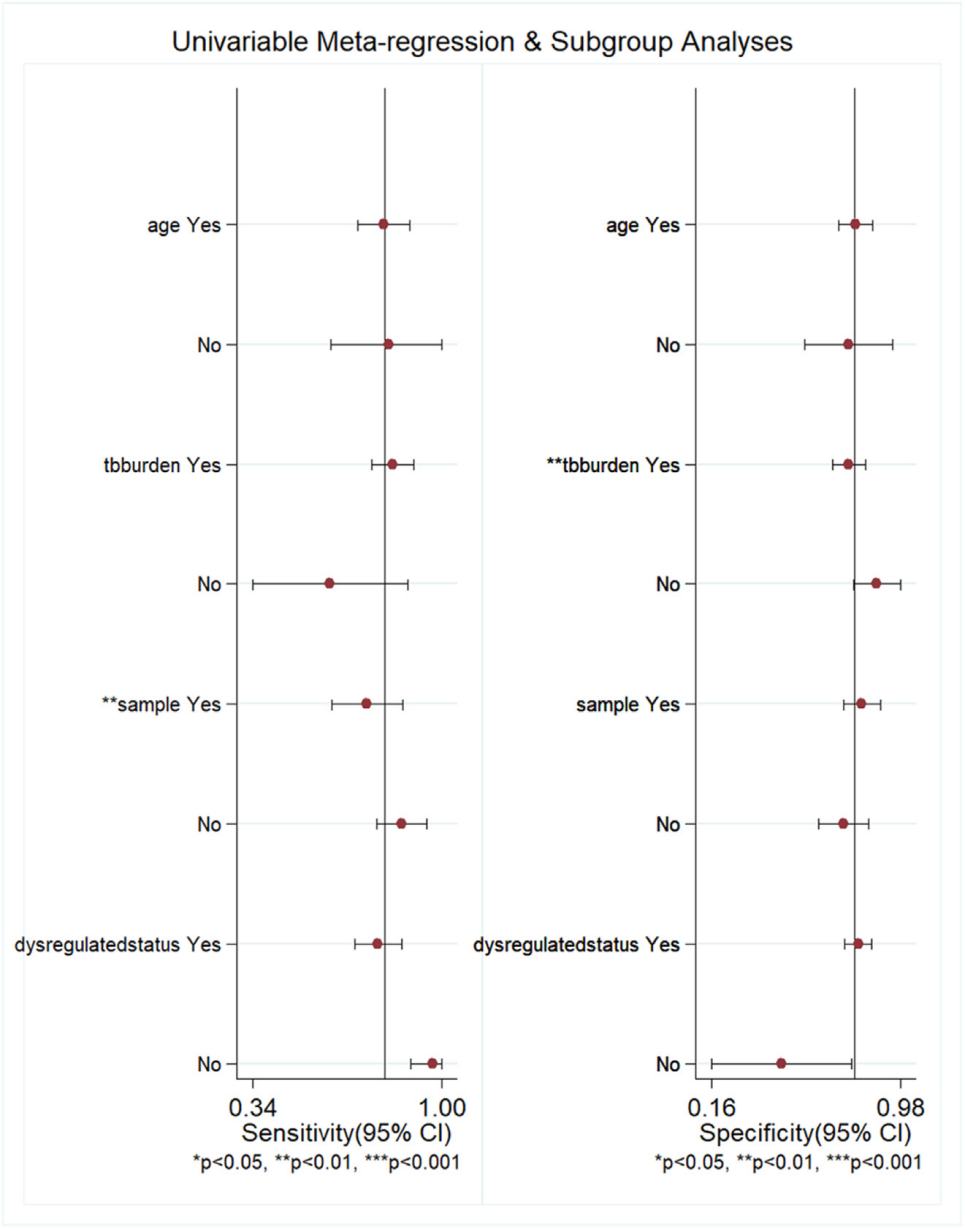
Meta-regression analysis results. For age, “yes” meant that participants were adults and “no” meant that participants were adults and children; for TB burden, “yes” meant that TB burden was high and “no” meant that TB burden was low; for sample, “yes” meant that the sample source was plasma and “no” meant that the sample source was not plasma; and for dysregulation status, “yes” meant that miRNA-29a was up-regulated and “no” meant that miRNA-29a was down-regulated.

The authors performed subgroup analysis of the included studies by age, TB burden, sample type, and miRNA-29a dysregulation status. The results showed that the sensitivity and specificity of miRNA-29a were similar in the two groups when stratified by age. The sensitivity of miRNA-29a was higher in areas with a high TB burden than in areas with a low TB burden, but the specificity was the opposite. Serum-derived miRNA-29a had the highest sensitivity, while plasma-derived miRNA-29a had the highest specificity. The results of the subgroup analysis are shown in Table 2. With the above 4 factors as covariables, meta-regression analysis was used to further explore the source of heterogeneity of the non-threshold effect, and the results are shown in Fig. 5. For sensitivity, the sample type had statistical significance (*p <* 0.01). For specificity, the TB burden was statistically significant (*p <* 0.01).

**Table 2:** Subgroup analysis results.

Subgroups	Studies (n)	Sensitivity	Specificity
Age			
Adult	11	0.78 (0.74‒0.80)	0.76 (0.73‒0.79)
Adult +child	2	0.82 (0.72‒0.89)	0.75 (0.63‒0.84)
TB burden			
High	11	0.84 (0.81‒0.86)	0.73 (0.69‒0.76)
Low	2	0.54 (0.46‒0.62)	0.89 (0.82‒0.93)
Sample type			
Plasma	7	0.72 (0.68‒0.76)	0.80 (0.76‒0.84)
Serum	3	0.90 (0.85‒0.93)	0.69 (0.63‒0.75)
Blood	3	0.78 (0.70‒0.84)	0.76 (0.65‒0.85)
Dysregulation status			
Up	12	0.77 (0.74‒0.80)	0.77 (0.74‒0.80)
Down	1	‒	‒

Dysregulated status: miRNA-29a was up-regulated or down-regulated in each study.

### Publication bias

**Fig. 6:**
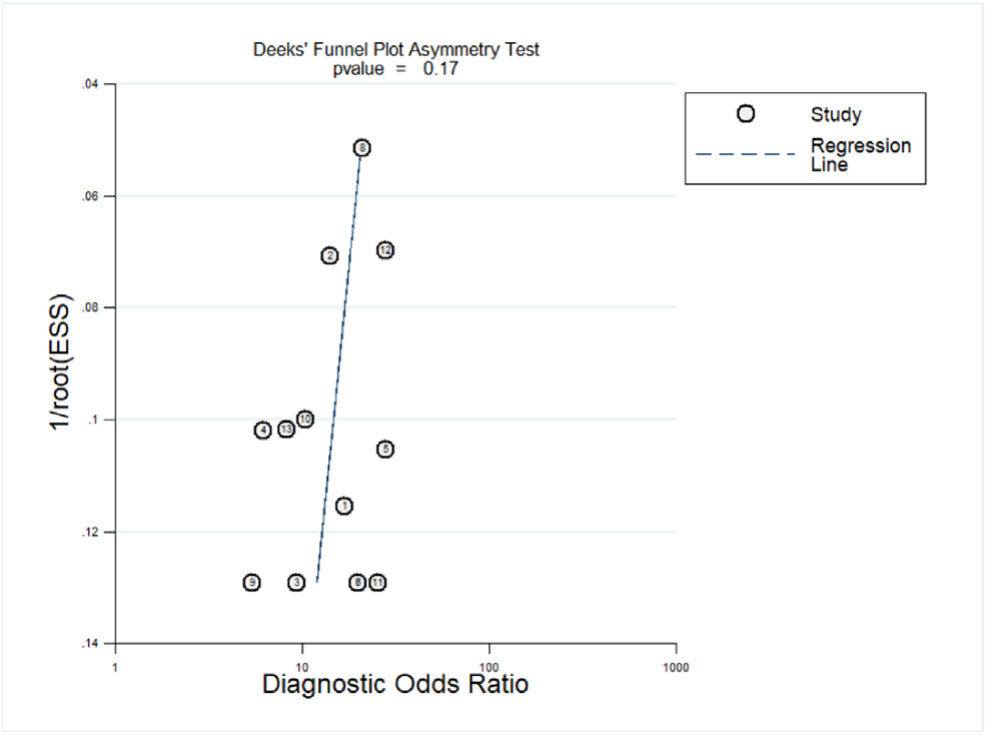
Deeks' funnel plots for the assessment of publication bias.

Deeks' funnel plot asymmetry was used to test for publication bias. As shown in Fig. 6, p = 0.17, *p* > 0.05, indicating that no significant publication bias was found in the included studies.

## Discussion

The traditional diagnosis method of TB is based on clinical and radiological evidence, but the confirmation test must be etiology and/or histology.^25^ Histological specimens usually require invasive procedures to obtain and are not easily obtainable in clinical practice. At present, there are three main effective methods for detecting active TB: microscopy, nucleic acid amplification tests, and cultures.^26^ The main limitations of microscopy are its low sensitivity and its inability to distinguish MTB from non-tuberculous mycobacteria.^27^ Nucleic acid amplification tests do not distinguish between viable and non-viable bacteria, and their detection depends on the bacterial load in the tested specimen.^28^ The culture of MTB from the analyzed sample is the definitive method of TB, but it usually takes two to six weeks.^29^ Despite many advances that have been made in the diagnosis of TB, accurate, fast, and simple diagnosis of active TB remains a challenge. miRNAs are involved in many normal cellular processes, such as cell cycle control, apoptosis, and developmental and physiological processes.^30^ In recent years, it has been found that miRNAs play important roles in regulating immunity against MTB.^31^ MTB can induce or inhibit the expression of miRNAs, and miRNAs are involved in regulating gene expression in the main target cells of MTB, such as macrophages, dendritic cells, natural killer cells, and T-cells.^32^ miRNA-29a is the most richly expressed member of the miRNA-29 family. It is a key regulatory factor of adaptive immunity. miRNA-29a is mainly located in the cytoplasm and is expressed in T-cells and B-cells as well as major helper cell types of thymic epithelial cells and dendritic cells. [33,34] miRNA-29a is a miRNA associated with MTB infection that inhibits the immune response to MTB by reducing IFN-γ levels.^20^

The effect of miRNA-29a in the diagnosis of active TB varies according to different study areas and subjects. Therefore, the authors summarized and analyzed the literature on the diagnosis of active TB with miRNA-29a and evaluated the value of miRNA-29a in the diagnosis of active TB. The present results showed that the combined sensitivity, specificity, PLR, NLR, and DOR of miRNA-29a in the diagnosis of active TB were 78 %, 76 %, 3.03, 0.27, and 14.03, respectively. The area under the SROC curve was 0.8564, indicating that miRNA-29a had a good ability to diagnose active TB.

The *I*^2^ of sensitivity and specificity were both greater than 50 %, suggesting significant heterogeneity in this study. The key to meta-analysis was to explore the source of heterogeneity. The Spearman correlation coefficient in this study was 0.730, *p* = 0.005, indicating that the threshold effect was one of the causes of heterogeneity. The results of sensitivity analysis showed that there was one study with discrete distribution, which may be one of the causes of heterogeneity. The authors further explored the source of heterogeneity of non-threshold effect by subgroup analysis and meta-regression. Subgroup analysis showed that the diagnostic sensitivity and specificity of miRNA-29a were similar in adults and non-adults. In high TB burden areas, miRNA-29a was more accurate in identifying TB patients, while in low TB burden areas, miRNA-29a was more accurate in identifying non-TB patients. Among different sample types, serum-derived miRNA-29a had the highest sensitivity, while plasma-derived miRNA-29a had the highest specificity. The results of regression analysis showed that the source of miRNA-29a samples and the burden of TB may also be the cause of heterogeneity. However, the present subgroup analysis did not fully cover the factors that may have contributed to the heterogeneity. Other factors such as HIV infection status may also explain the heterogeneity, which unfortunately was not analyzed due to insufficient data.

The limitations the our meta-analysis were that most of the included studies were conducted in countries with a high burden of TB, and the sample size for active TB was only 872 in the 13 included articles. Therefore, a larger study with uniform standards is needed to verify the present results in the future.

## Conclusion

The results of this meta-analysis indicated that miRNA-29a had a good diagnostic performance as a biomarker for the diagnosis of active TB.

## Ethics approval and consent to participate

Not applicable.

## Funding

No funding was received.

## CRediT authorship contribution statement

**Xiaoying Li:** Project administration, Methodology, Data curation, Formal analysis, Writing – original draft. **Yuehong Xu:** Methodology, Data curation, Formal analysis, Writing – review & editing. **Pu Liao:** Project administration, Writing – review & editing.

## Conflicts of interest

The authors declare no conflicts of interest.
